# IDS for Industrial Applications: A Federated Learning Approach with Active Personalization

**DOI:** 10.3390/s21206743

**Published:** 2021-10-11

**Authors:** Vasiliki Kelli, Vasileios Argyriou, Thomas Lagkas, George Fragulis, Elisavet Grigoriou, Panagiotis Sarigiannidis

**Affiliations:** 1 Department of Electrical and Computer Engineering, University of Western Macedonia, 501 31 Kozani, Greece; vkelly@uowm.gr (V.K.); gfragulis@uowm.gr (G.F.); psarigiannidis@uowm.gr (P.S.); 2 Department of Networks and Digital Media, Kingston University, London KT1 1LQ, UK; Vasileios.Argyriou@kingston.ac.uk; 3 Department of Computer Science, Kavala Campus, International Hellenic University, 654 04 Kavala, Greece; 4 Sidroco Holdings Ltd., Nicosia 1077, Cyprus; egrigoriou@sidroco.com

**Keywords:** IoT, IDS, critical infrastructure, federated learning, machine learning, active learning, personalization

## Abstract

Internet of Things (IoT) is a concept adopted in nearly every aspect of human life, leading to an explosive utilization of intelligent devices. Notably, such solutions are especially integrated in the industrial sector, to allow the remote monitoring and control of critical infrastructure. Such global integration of IoT solutions has led to an expanded attack surface against IoT-enabled infrastructures. Artificial intelligence and machine learning have demonstrated their ability to resolve issues that would have been impossible or difficult to address otherwise; thus, such solutions are closely associated with securing IoT. Classical collaborative and distributed machine learning approaches are known to compromise sensitive information. In our paper, we demonstrate the creation of a network flow-based Intrusion Detection System (IDS) aiming to protecting critical infrastructures, stemming from the pairing of two machine learning techniques, namely, federated learning and active learning. The former is utilized for privately training models in federation, while the latter is a semi-supervised approach applied for global model adaptation to each of the participant’s traffic. Experimental results indicate that global models perform significantly better for each participant, when locally personalized with just a few active learning queries. Specifically, we demonstrate how the accuracy increase can reach 7.07% in only 10 queries.

## 1. Introduction

Machine learning solutions currently have universal utilization in IoT applications [1,2,3]. Specifically, machine learning helps to extract insights and knowledge from IoT data, attributes which would have been extremely difficult to obtain with other means [4]. For this purpose, machine learning has been successfully applied in multiple areas, from AI-enabled assistants [5] and speech recognition [6], to the time-critical industrial sector [7,8]. In addition, with the help of machine-learning-enabled solutions, robust IDS can be created and applied for rapid and accurate detection of malicious attempts against the network [9,10,11].

Attacks against the industrial sector, as indicated from past incidents, can have severe consequences. Such incidents include the December 2015 cyberattack against Ukraine’s power grid, which resulted in complete electricity disruption for 225,000 people [12,13]. In addition, as Stuxnet, the first known cyber warfare weapon [14,15], indicated, nuclear power plants have also been targeted by cyberattacks, thus emphasizing the urgent need for adequate security measures in such critical domains. As such, the adoption of security measures, such as IDS for rapid attack detection, is necessary to ensure safe and secure operations.

Machine learning for the creation of IDS is not a new concept, as intelligent solutions can boost the efficiency of IDS. However, creating IDS with multiple nodes, characterized by differences in traffic, is not an easy task. Traditional centralized solutions assume a central server, receiving IoT data [16] and utilizing them to train models capable of distinguishing regular traffic from attack attempts [17]. Such solutions consume network resources, as massive data from all IoT devices would have to be sent to the central server [18]. Furthermore, such solutions raise issues with data privacy [19] and single-point-of-failure concerns [20]. As data would flow from the devices to the server for training, data loss is a possibility, as well as data tampering or false data injection from a malicious entity.

Such issues are addressed with Federated Learning (FL). FL is a technique that requires model updates to be sent to the server, while data remain locally on each device, thus ensuring data privacy during model training [21]. However, traffic from multiple IoT devices may not be characterized by the same attributes. As such, final model personalization methods are required. Notably, dataset labelling is an expensive and time-consuming process, especially regarding large datasets composed by IoT devices [22]. Active Learning (AL) solutions have emerged to tackle such limitations, as the learner can choose the samples to learn from  [23], thus, making this technique excellent for model personalization.

The purpose of this paper is three-fold:

- present a 2-stage methodology for pairing FL and AL strategies, with the former offering distributed, secure and private global model training as the first training stage, and the latter for improving the generated model’s performance, as the last training stage.
- analyse and compare the amount of annotating effort, or, AL queries needed to achieve a sufficiently better, customized local model.
- design and implement an attack detection and classification model based on DNNs, with the utilization of DNP3-specific attacks, transformed into flow-based traffic representations, serving as a training set.

The rest of the paper is structured as follows. In Section 2, related previous work is explained. Then, in Section 3, our proposed methodology is presented, and is described in detail through Section 3.2, Section 3.3 and Section 3.4. Section 4 indicates our experimental process and the results obtained by applying our methodology, and finally, Section 5 concludes this paper.

## 2. Previous Work

Currently, data privacy is one of the focal research points, especially due to the General Data Protection Regulation (GDPR) adopted by the European Union [24]; thus, federated learning has gained a lot of attention for allowing distributed model training without local data exchange [25]. A lot of research has been conducted with regards to the application of federated learning for creating IDS. The authors in [26] propose a federated training approach, on Gated Recurrent Units (GRUs) models, to detect anomalies in IoT networks, in order to timely recognize intrusion attempts. Similarly, the authors in [27] target the insufficiency of current IDS by proposing DÏoT, an autonomous self-learning system capable of detecting compromised IoT devices, without needing a human to intervene in the process, or labeled datasets. Specifically, DÏoT detects anomalies in devices’ communication, by aggregating behavior profiles with the utilization of the federated learning approach. Federated learning is also utilized for the creation of an IDS catering to the needs of Medical Cyber-Physical Systems (MCPS), where patients are clustered based on their profiles, and each cluster develops its own federated model according to the input that is received by the registered patients. If any abnormality is detected due to a malicious intervention such as data modification or injection attack, alerts are generated [28].

As noted, active learning reduces the amount of labeled samples required for model training, by locating query-worthy samples to be learn from. The integration of this methodology for detecting attacks has been researched in the past. Specifically, active learning for network intrusion detection can be seen as an unsupervised task according to [29]. Furthermore, the authors propose a novel querying strategy to reduce labelling effords. Experimental results indicated that the ActiveSVDDs were able to distinguish normal and attack data, while reducing labelling actions. The authors in [30] present a method of reducing outlier detection to a classification problem by representing outliers using artificially generated examples, and later applying active learning for selective sampling. According to experiments conducted, the proposed methodology yields better results than methods which apply the same reduction, but use regular classification procedures. The authors in [31] suggest building active learning procedures on top of deep learning solutions for unsupervised anomaly detection. This is achieved by adding an Unsupervised to Active Inference (UAI) layer on top of unsupervised deep learning architectures. Experimental results showed that models were able to achieve similar or improved results than their non-active learning enhanced counterparts.

As noted, a lot of great research has been conducted for finding solutions for private, distributed model training and active learning for anomaly detection and classification. As such, we aim to further contribute in the aforementioned research areas, specifically by combining federated learning, active learning and Deep Neural Network (DNNs) strategies to enhance data privacy, and introduce personalization methods in a semi-supervised approach in order to create attack classification-based IDS.

## 3. Methodology

As described in Section 2, a lot of research has been conducted in order to identify optimal methods for cyberattack detection and classification, aiming in the creation of robust IDS, especially for the critical industrial sector where rapid and precise attack detection is of essence. An important aspect for consideration while training classification models for application on each device on the network, is the difference in traffic attributes. As such, personalization methods should be applied in order to ensure that models running on each device cover a plethora of attack cases, while also being customized to the devices’ needs.

### 3.1. Overall Description

The proposed methodology provides a data privacy-friendly approach for training a DNN on attack detection and classification, while adapting the final model to the requirements of each device, in order to produce accurate, and personalized results. In the methodology proposed, to ensure that local data would never leave the device and thus reducing the amount of messages constrained devices would have to communicate, while simultaneously addressing the issue of training data tampering, the multi-class classification DNN model training based on various attack scenarios was conducted with FL. After the FL process comes to a halt, the global model is personalized with AL, by each of the participating devices. The proposed methodology is divided into two stages:

1. the FL global model training and
2. the personalisation stage using AL.

**Figure 1:**
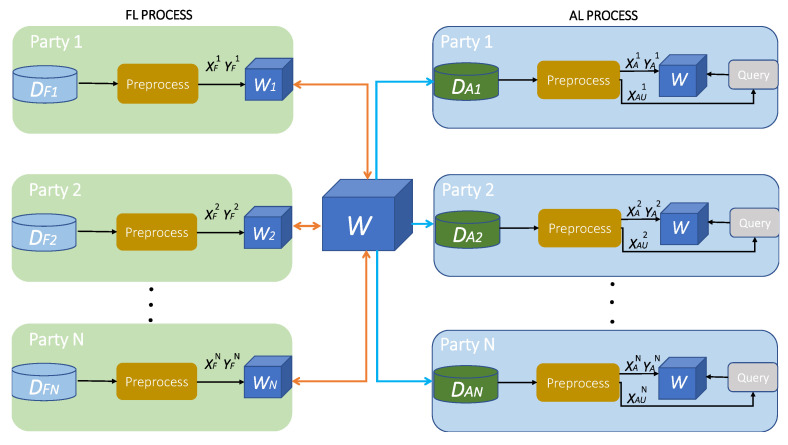
The proposed methodology combining FL for global model formation and AL for model personalization.

Figure 1 below, represents the entire methodology of this paper.

The entire machine learning process is divided into two stages, the FL and the AL stage. We assume *N* participating entities, where each party p∈[1,N] holds locally two inputs, the $D_{F_{p}}$ input used for training the attack detection and classification model in federation with the rest of the participants, and the $D_{A_{p}}$ input used for adapting the final global federated model to each of the participants’ traffic. During the FL stage, each party *p* pre-processes the $D_{F_{p}}$ inputs by applying feature normalization to turn data into values in the [0,1] range, as demonstrated in (1) [32] where $x_{sc}$ is the scaled feature value, x is the feature vector and $x_{i}$ is the initial feature value;

(1) $$x_{sc}=\frac{x_{i}-\min\left(x\right)}{\max(x)-\min(x)}$$

then, the resulting inputs which are transformed in $X_{F}^{p}$ data points and $Y_{F}^{p}$ labels, are fed into the DNN in order to train the attack detection and classification model via supervised learning. As a result of the training procedure each *p* obtains the updates $W_{p}$, and via aggregating the results from each *p*, the global model *W* is formed. After the FL stage concludes, the AL is initiated, during which, each *p* divides the $D_{A_{p}}$ input into two parts and transforms the first part into $X_{A}^{p}$ data points, via normalization, (1) and $Y_{A}^{p}$ corresponding labels while transforming the latter part, also via normalization (1), into the $X_{\mathrm{AU}}^{p}$ sampling pool, containing only unlabeled data points. *W* is further trained in a supervised manner by each *p* using the $X_{A}^{p}$ data points and the $Y_{A}^{p}$ labels; then, by following a querying strategy, the model selects the most informative samples from the data pool $X_{\mathrm{AU}}^{p}\neq X_{A}^{p}$. When the active training process concludes, each *p* has formed the final, personalized attack detection and classification model.

In the sections below, the FL and AL stage are explained in detail, while the attack detection and classification model is presented.

### 3.2. Federated Learning for Cyber-Attack Detection

We consider the scenario where network traffic data containing normal and malicious records, is located in various devices. Specifically, the *p*-th device, or party, p=1,…,N∈N, has $\left(X_{F}^{p},Y_{F}^{p}\right)\in D_{F_{p}}$ local database, containing $l_{p}$ data points, given as

(2) $$X_{F}^{p}=\left(\begin{array}{c} x_{F_{1}}^{p}\\ \vdots \\ x_{F_{l_{p}}}^{p} \end{array}\right),Y_{F}^{p}=\left(\begin{array}{c} y_{F_{1}}^{p}\\ \vdots \\ y_{F_{l_{p}}}^{p} \end{array}\right)$$

where a label $y_{F_{k}}^{p}\in R$, with $k\in [1,\ldots ,l_{p}]$, is associated with each training data point $x_{F_{k}}^{p}\in R$. Each device *p* uses its database $D_{F_{p}}$ to train a local model, represented by vector $W_{p}$. Training is carried out to minimize a local objective $f(W;D_{F_{p}})$, based on a loss measure L(·) [33], where W represents the global model’s vector. The local objective for *p* is given by:

(3) $$f\left(W;{\left\{x_{F_{k}}^{p},y_{F_{k}}^{p}\right\}}_{k=1}^{l_{p}}\right)=\frac{1}{l_{p}}\sum_{k=1}^{l_{p}}L\left(x_{F_{k}}^{p},y_{F_{k}}^{p},W\right)$$

Thus, the objective of every *p* is to obtain the parameters $W_{p}$ which minimize (3):

(4) $$W_{p}=\underset{W}{arg min}f\left(W;D_{F_{p}}\right)$$

An aspect for consideration while training models with federated learning, is the fusion technique used by the central aggregator, to combine model updates coming from multiple participants *p*. According to the iterative averaging approach, the server requests local model updates $W_{p}^{r}$ from parties *p* at each federated round *r*, and then the averaging aggregation is performed over the collected models’ weights, where the global model $W^{r}$ is updated by the mean of all the collected local models’ weights, like so:

(5) $$W^{r}=\frac{W_{p}^{r}+W_{p+1}^{r}+\ldots +W_{N}^{r}}{N}$$

The federated learning procedure combines local training described by (4) and global aggregation and fusion, described by (5) in a set of iterative steps, followed until the desired convergence is achieved, without having parties share their local database. Specifically, at each round *r*:

(1) The server sends the global model $W^{r}$ to the participants, and each *p* sets their local model to be the global model $W_{p}^{r}=W^{r}$.
(2) Each party *p* updates the model from $W_{p}^{r}$ to $W_{p}^{\left(r+1\right)}$, based on (4), by utilizing their local database $D_{F_{p}}$.
(3) The participants send their locally calculated updates back to the server for global model formation, according to (5).

In Algorithm 1 below, the federated process is described.
**Algorithm 1** FL Stage1:Aggregator Side2:**for***r***do**3:      **for** p=1,2,…,N **do**4:            Send $W^{r}$ to *p*5:    **end for**6:    **for** p=1,2,…,N **do**7:         $W^{r}$ += Request $W_{p}^{\left(r+1\right)}$ from *p*8:    **end for**9:    $W^{\left(r+1\right)}\leftarrow \frac{W^{r}}{N}$10:**end for**11:Worker Side12:$W_{p}^{r}=W^{r}$13:**for**epochs**do**14:      $W_{p}^{\left(r+1\right)}$← Train $W_{p}^{r}$ with $\left(X_{F}^{p},Y_{F}^{p}\right)\in D_{F_{p}}$15:**end for**16:On Request Send $W_{p}^{\left(r+1\right)}$ to Aggregator

### 3.3. Attack Detection and Classification Model

**Figure 2:**
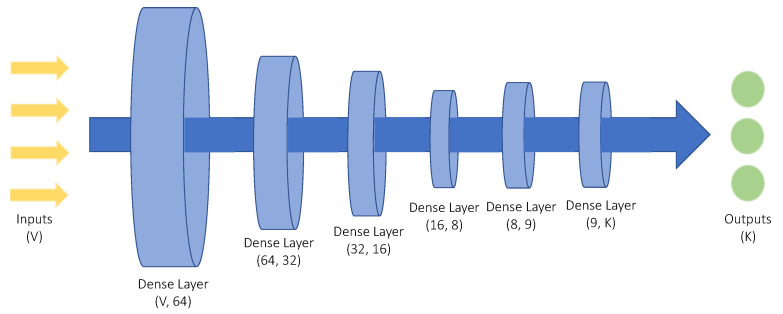
The proposed DNN architecture, receiving *V* features as an input and producing *K* outputs.

DNNs are powerful machine learning tools, utilized in problems with high complexity. As such, the attack detection and classification model implemented in this paper, follows a DNN architecture. Specifically, the various layers composing the DNN, can be observed in Figure 2. The classification model is compiled with Categorical Crossentropy (6), a loss function suitable for classification problems where *K* denotes the number of classes, $b_{kc}$ is a binary indicator that detects whether the *k*th input belongs to the *c* category, while the output $o_{kc}$ denotes the predicted probability for the *k*th input to belong to the *c* category. Finally, the optimization algorithm used was Adam with a learning rate of 0.001.

(6) $$L_{CCE}=-\sum_{c=1}^{K}b_{kc}log\left(o_{kc}\right)$$

The architecture of the DNN, as observed in Figure 2, consists of 6 layers, all of which are Dense. The first layer takes as an input a *V* number of features, while it consists of 64 neurons. The next 3 layers have a decreasing number of neurons, while the 5th layer consists of 9 neurons. Finally, the output layer has *K* neurons, where *K* denotes the number of classes. All layers but the output one, are activated by the ReLu activation function (7) with *x* denoting the input value:

(7) $$f\left(x\right)=\left\{\begin{array}{cc} 0 & \mathrm{if}\;x\leq 0\\ x & \mathrm{if}\;x>0 \end{array}\right.$$

The last layer is activated by the Softmax activation function (8), utilized in multi-class classification problems, which turns input values to probabilities. Specifically, for each output of the last layer, Softmax provides a probability distribution of class membership. This is achieved by dividing the exponential value of output $z_{i}$ with the summation of all exponentials:

(8) $$SM\left(z_{i}\right)=\frac{e^{z_{i}}}{\sum_{j=1}^{K}e^{z_{j}}}$$

### 3.4. Active Learning

Active Learning is a semi-supervised machine learning approach which addresses the difficulties of adding manually labels to an unlabeled dataset, by dynamically choosing samples and querying an oracle for the provision of labels. Initially, the learner located in each party *p*, is trained on a set of fully labeled samples, $\left(X_{A}^{p},Y_{A}^{p}\right)\in D_{A_{p}}$, containing $d_{p}$ data points, given as:

(9) $$X_{A}^{p}=\left(\begin{array}{c} x_{A_{1}}^{p}\\ \vdots \\ x_{A_{d_{p}}}^{p} \end{array}\right),Y_{A}^{p}=\left(\begin{array}{c} y_{A_{1}}^{p}\\ \vdots \\ y_{A_{d_{p}}}^{p} \end{array}\right)$$

where, a label $y_{A_{k}}^{p}\in R$, with $k\in [1,\ldots ,d_{p}]$, is associated with each training data point $x_{A_{k}}^{p}\in R$.

After the first round of training, the learner gets introduced to a pool of un-annotated samples, $X_{\mathrm{AU}}^{p}\in D_{A_{p}}\neq X_{A}^{p}$, containing $z_{p}$ data points:

(10) $$X_{\mathrm{AU}}^{p}=\left(\begin{array}{c} x_{{\mathrm{AU}}_{1}}^{p}\\ \vdots \\ x_{{\mathrm{AU}}_{z_{p}}}^{p} \end{array}\right)$$

Following a querying strategy, the learner selects the most informative, or the most uncertain instance $x_{\mathrm{AUi}}^{p}\in X_{\mathrm{AU}}^{p}$, with $i\in [1,\ldots ,z_{p}]$ and poses a query to the handler in order to be informed about the corresponding label $y_{AUi}^{p}$. The learner, expands its knowledge, having obtained the $y_{AUi}^{p}$ to the queried $x_{\mathrm{AUi}}^{p}$. This process reiterates until a preferred accuracy is achieved. An example of the aforementioned AL process is represented in Algorithm 2, below.
**Algorithm 2** AL Stage1:*p* Side2:Initial adaptation of *W* with $\left(X_{A}^{p},Y_{A}^{p}\right)\in D_{A_{p}}\to learner$3:**for**iter=1,2,…,R**do**4:       Select $x_{\mathrm{AUi}}^{p}\in X_{\mathrm{AU}}^{p}$5:       Label Query $x_{\mathrm{AUi}}^{p}\to y_{AUi}^{p}$6:       Train learner with $(x_{\mathrm{AUi}}^{p},y_{AUi}^{p})\to personalizedmodel$7:**end for**

The querying strategy utilized successfully in multiple scenarios, namely Uncertainty Sampling, emphasizes on selecting unlabeled samples which the learner is mostly uncertain about. Several measures can be used for this, one being classification uncertainty defined in (11), where $x_{\mathrm{AUk}}^{p}$ is the instance to be predicted and $py_{AUk}^{p}$ is the most likely prediction probability for this instance:

(11) $$S\left(x_{\mathrm{AUk}}^{p}\right)=1-P\left(py_{AUk}^{p}|x_{\mathrm{AUk}}^{p}\right)$$

In order to pick the most informative instance $x_{\mathrm{AUi}}^{p}$, the learner aims to choose a sample amongst $X_{\mathrm{AU}}^{p}$ for which the classification uncertainty *S* is the highest (12).

(12) $$x_{\mathrm{AUi}}^{p}=\underset{x_{\mathrm{AUk}}^{p}\in X_{\mathrm{AU}}^{p}}{arg max}S\left(x_{\mathrm{AUk}}^{p}\right)$$

As such, AL employs statistical analysis to ensure that the most informative data points are selected for labelling from a pool of samples, thus minimizing the annotation efforts, and providing a cost-effective solution for training machine learning models.

## 4. Results

The proposed approach for collaborative model training and customization, is divided into the two machine learning stages explained in Section 3 above, namely the FL stage described in Section 3.2 to preserve worker data privacy, and the AL stage described in Section 3.4 for global model personalization based on each of the participant’s needs. We consider that each party’s local database, containing network traffic data is characterized by different attributes. Thus, to ensure its suitability, the global model is personalized and adapted to each participant’s communication characteristics.

The database used for distributed model training and personalization consists of network traffic data in the form of network flows. The protocol used in the experiments in DNP3, a protocol widely used in industrial settings; DNP3 assumes a central master node directing and requesting data from multiple slave nodes, which in turn handle and respond to the master’s requests. For experimental purposes, normal DNP3 communication was simulated, while attacks were conducted against the simulated infrastructure to gather malicious packets. Specifically, the attacks were either DNP3-specific, targeting the protocol’s vulnerabilities, or generic. DNP3-specific attacks included scanning for DNP3 ports with nmap DNP3-centered scripts, like DNP3 enumerate and DNP3 info, malicious cold and warm restart requests crafted to restart the slaves, packets created with the purpose of damaging slaves’ data by re-initializing their local database, attacks directing the slaves to cease DNP3 applications with the stop application attack, and ordering slaves to disable their ability to send unsolicited responses, thus making them unable to notify the master in case of abnormalities. The replay attack was performed as a generic malicious attempt, aiming to replay or delay the transmission of a normal packet.

Network packets containing malicious and normal traffic of DNP3, were captured and processed into DNP3-specific network flows, consisting of 100 features, centered around the protocol’s attributes such as MostCommonREQ FUNC CODE, referring to the most common DNP3 function code used in the DNP3 master’s requests, DeviceRestartFragment, which counts the DNP3 slave’s responses indicating a restart, different DNP3 layers payload size, etc.; in addition, general network traffic features are present in each flow, such as packet inter-arrival times, flow bytes/sec, etc. Each flow was utilized as the input to the model, while the corresponding label, describing the nature of the flow, is considered as the desired output of the model. As this is an attack detection and classification problem, the labels were classified in a total of 9 classes, describing the attack performed, or a normal flow state. The goal of the machine learning process is to develop models trained to recognize a variety of attacks, without having to share data with the server, while adapting the final model to the participants’ requirements.

**Figure 3:**
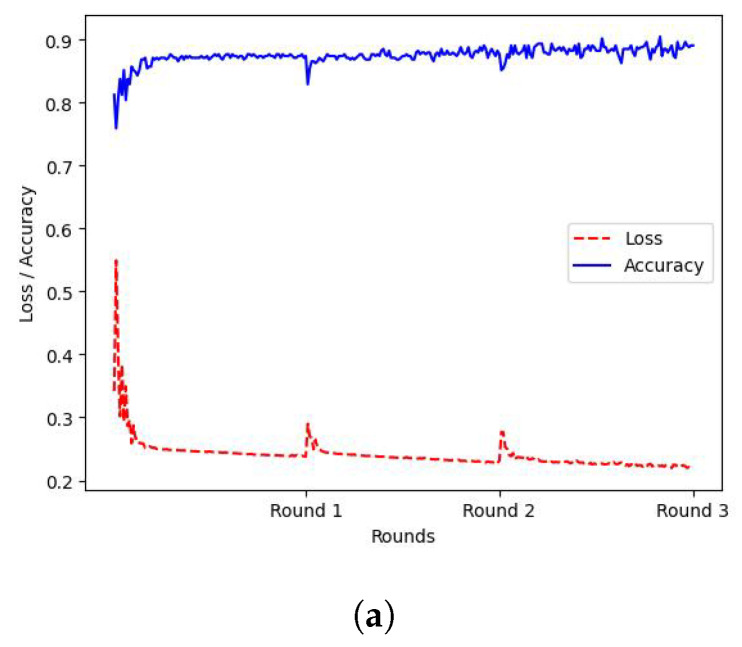

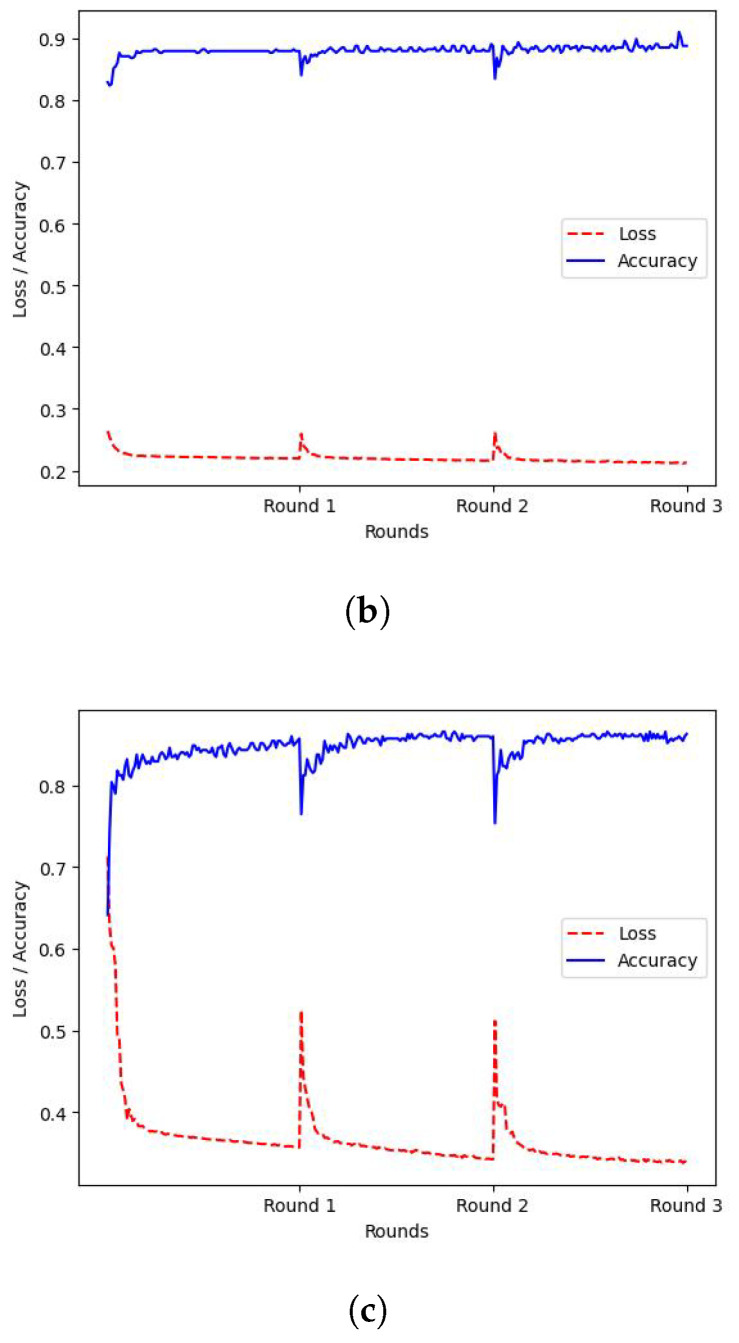
FL Training. *X*-axis: Federated Rounds, *Y*-axis: value of accuracy(blue and loss (red). (**a**) Worker 1 (W1) FL Training Metrics; (**b**) Worker 2 (W2) FL Training Metrics; (**c**) Worker 3 (W3) FL Training Metrics.

Initially, the FL approach described in Section 3.2 was applied, to train models in *r* = 3 consecutive federated rounds. Specifically, p=[1,2,3], or 3 workers were deployed for distributed training, each one holding locally FL datasets $D_{F_{1}},D_{F_{2}},D_{F_{3}}$ containing instances from all 9 classes, however, for each of the workers, the FL dataset was biased towards a specific class by 50%. The training loss and accuracy of the FL procedure for each worker can be observed in Figure 3, where the X-axis represents the federated rounds, while the Y-axis represents the corresponding value of the accuracy or loss.

After the FL training concludes, each worker obtains an identical global model, created by the server, who fuses local model updates using Equation (5). At this point, the workers measure how well the global model is able to perform, by utilizing their local validation set. Each worker’s validation set, shows a 30% bias towards the same class as the dataset used during the FL procedure. In order to measure how well the model performs, the accuracy, precision and F1 scores where used, as described in Equations (13)–(15) respectfully, where TP is the number of true positives, TN is the number of true negatives, FP is the number of false positives and FN is the number of false negatives classified by the model. The aforementioned results can be observed in Table 1.

(13) $$accuracy=\frac{TP+TN}{TP+TN+FP+FN}$$

(14) $$precision=\frac{TP}{TP+FP}$$

(15) $$F1=\frac{TP}{TP+\frac{1}{2}\left(FP+FN\right)}$$

The next training step for each worker refers to the application of AL to further train the global model with local inputs, thus customizing it to each of the workers’ traffic. The AL step was divided into 4 sub-experiments, in order to measure the final local models’ performance under multiple local dataset balance scenarios. To this end, the AL inputs for each worker, were divided into the following categories, and models resulting from each category where evaluated:

(1) 20% Bias, towards the same class as FL
(2) 50% Bias, towards the same class as FL
(3) 70% Bias, towards the same class as FL
(4) No Bias (Equal number of class instances)

As previously mentioned, dataset annotation is an expensive and time-consuming process. To this end, we assume a budget of maximum 40 queries answered during the AL sampling process, per local dataset, to keep the labelling effords to a minimum, while still offering model adaptation. Thus, for each category mentioned above, models were evaluated after AL training with 10, 20, 30 and 40 queries.

### 4.1. Category 1: 20% AL Bias

**Figure 4:**
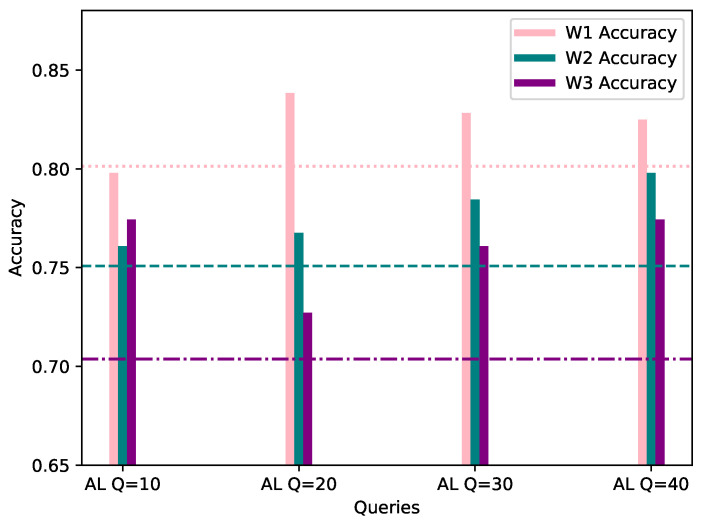
Accuracy of W1, W2 and W3’s models personalized with 20% AL dataset bias (*Y*-axis) per Query (*X*-axis), evaluated using their corresponding evaluation datasets. The FL accuracy for each worker is represented by the horizontal line of the worker’s respective color.

This category assumes a 20% bias of the local AL dataset, towards the same class as the worker’s FL dataset. In order to provide a fair comparison, the evaluation process was conducted with the same data as FL, and the same evaluation methods. In Table 2, the accuracy, precision and F1 score of the AL process for each workers’ local model is shown, while Figure 4 visualizes the accuracy score, per number of queries, with the workers’ corresponding FL score considered as the starting point.

**Figure 5:**
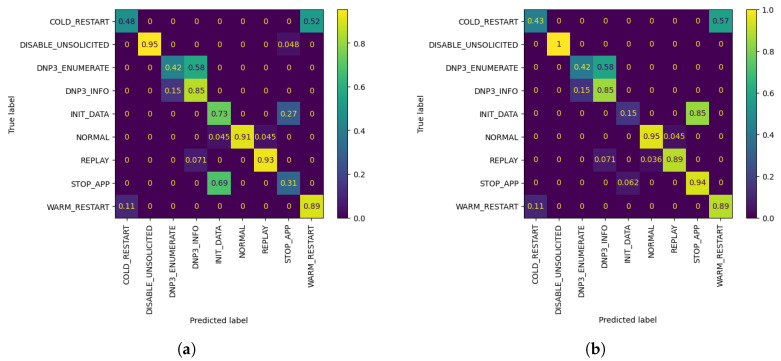
W1 Confusion Matrices. (**a**) W1’s evaluation of global FL model after r=3 using its evaluation dataset; (**b**) W1’s evaluation of customized model by AL, after Q = 10 queries, using its evaluation dataset.

As observed in the underlined results in Table 2 and Figure 4 above, the metrics show an increase when compared to the corresponding FL metrics in Table 1 in the vast majority of cases. It is worth noting that, even in cases where the metrics show a minor decrease, such as W1 AL model’s accuracy with 10 queries, the model is able to classify correctly all of the network flows which belong to W1’s biased class, namely DISABLE UNSOLICITED, whereas the global federated model showed inability to do so. The confusion matrices resulting by W1’s FL and AL evaluation for 10 queries can be observed in Figure 5. In addition, through Figure 4 it is becomes clear that for W1 and W3, 10–20 queries suffice for increasing the accuracy of their local model, while for W2, training can be stopped after 10–20 queries in case of a strict budget, as there is still improvement in accuracy. Specifically, for W3 the improvement is massive compared to W1 and W1, as its accuracy increased by 7.07% in only 10 AL queries.

### 4.2. Category 2: 50% AL Bias

**Figure 6:**
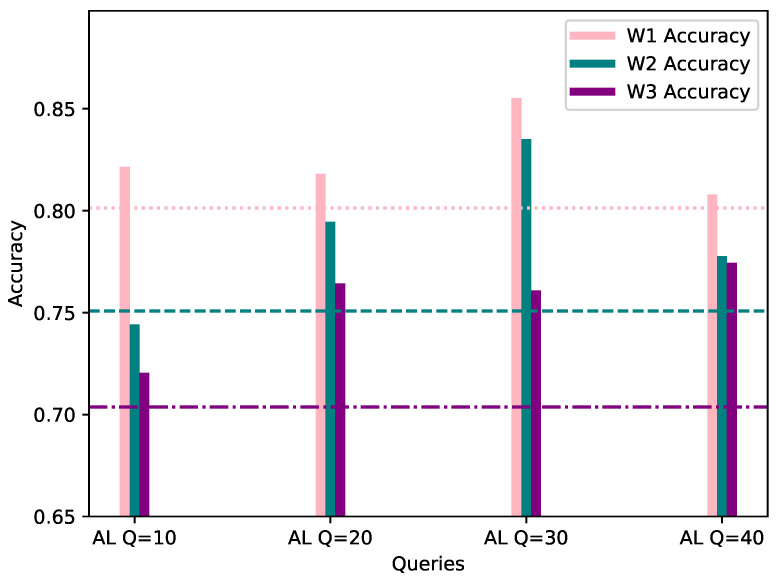
Accuracy of W1, W2 and W3’s models personalized with 50% AL dataset bias (*Y*-axis) per Query (*X*-axis), evaluated using their corresponding evaluation datasets. The FL accuracy for each worker is represented by the horizontal line of the worker’s respective color.

This category assumes a 50% bias of the local AL dataset, towards the same class as the worker’s FL dataset; similarly to Category 1’s results, the evaluation process was conducted with the same data as FL, and the same evaluation methods are applied. The results are depicted in a similar manner, with the higher metric value highlighted in Table 3 and the accuracy shown in Figure 6 below.

**Figure 7:**
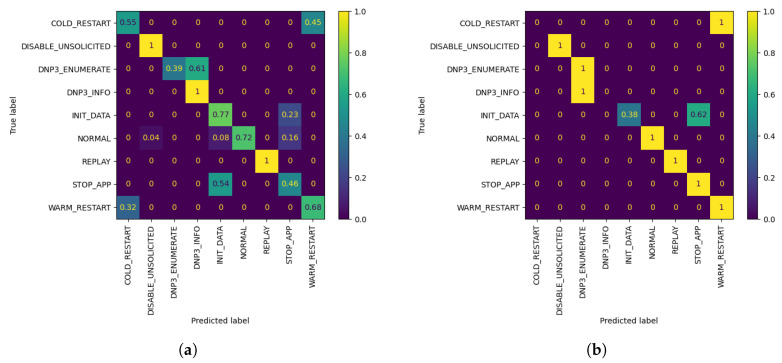
W3 Confusion Matrices. (**a**) W3’s evaluation of global FL model after r=3 using its evaluation dataset; (**b**) W3’s evaluation of customized model by AL, after Q = 10 queries, using its evaluation dataset.

It is observed that W3 shows a drop in Precision and F1 scores, although the overall accuracy is improved by training with AL, compared with standalone FL. However, W3 is able to classify correctly all validation samples which belong to the biased class, namely WARM RESTART, with only 10 queries, when the FL global model is unable to perform as well. This can be seen in Figure 7, which depicts the confusion matrices of W3’s evaluation of the FL and AL model with 10 queries. Furthermore, for 50% biased local database, models seem to peak in accuracy with 30 queries for W1 and W2, with the increase being 5.39% for the former and 8.42% for the latter, while W3 shows significant accuracy increase of 6.06% after 20 queries.

### 4.3. Category 3: 70% AL Bias

**Figure 8:**
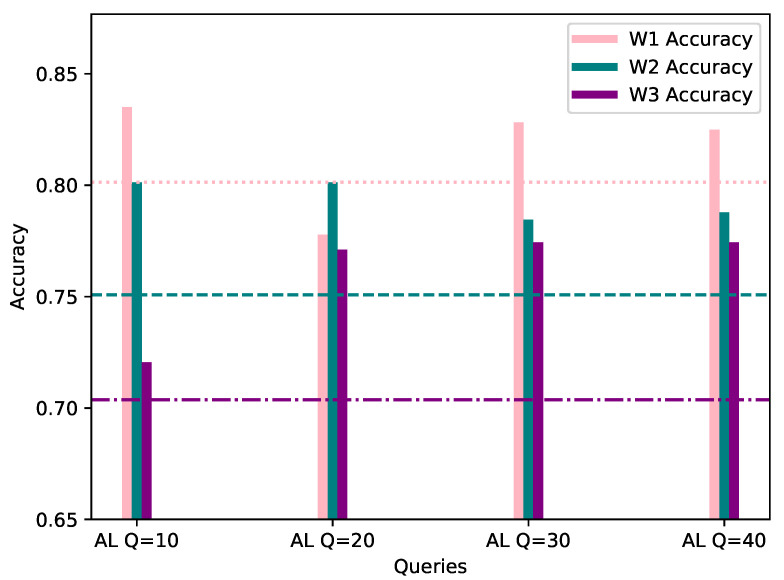
Accuracy of W1, W2 and W3’s models personalized with 70% AL dataset bias (*Y*-axis) per Query (*X*-axis), evaluated using their corresponding evaluation datasets. The FL accuracy for each worker is represented by the horizontal line of the worker’s respective color.

Similarly to the previous categories, Category 4 supposes a 70% bias of the local AL dataset, towards the same class as the worker’s FL dataset. The evaluation process was conducted with the same data as FL, and the same evaluation methods. The results of AL training with 70% biased datasets, are depicted in Table 4 and Figure 8 below.

**Figure 9:**
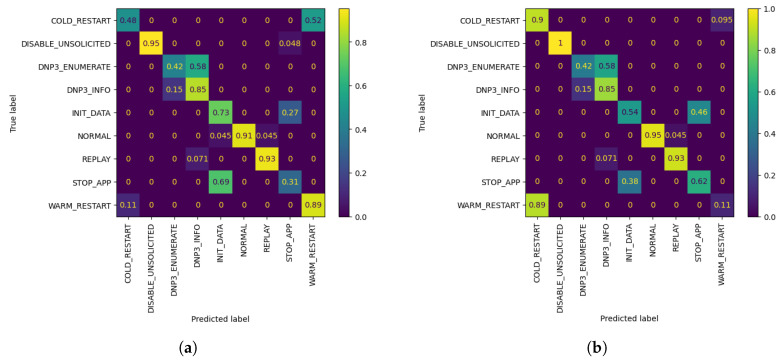
W1 Confusion Matrices. (**a**) W1’s evaluation of global FL model after r=3 using its evaluation dataset; (**b**) W1’s evaluation of customized model by AL, after Q = 20 queries, using its evaluation dataset.

From Table 4, it is observed that Worker 1’s customized model with 20 queries shows lower metric values when evaluated against the FL global model. However, the AL model is able to predict correctly all of the class instances which belong to the biased class category, namely DISABLE UNSOLICITED. This can be validated through the confusion matrices shown in Figure 9, proving that the personalized model is able to perform better when taking as an input an instance which better describes the worker’s dataset. Moreover, local models perform significantly better in terms of accuracy with only 10 queries for W1 and especially W2, with the former showing improved accuracy of 3.37% and the latter of 5.05% as seen in Figure 8.

### 4.4. Category 4: Balanced AL

The final experiment assumes a fully balanced AL dataset, and follows the same evaluation process are the categories above. The results can be observed in Table 5 below.

**Figure 10:**
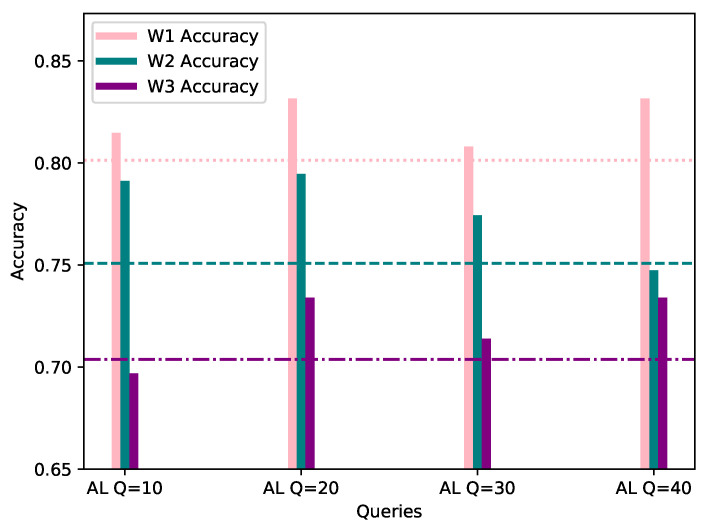
Accuracy of W1, W2 and W3’s models personalized with balanced AL dataset (*Y*-axis) per Query (*X*-axis), evaluated using their corresponding evaluation datasets. The FL accuracy for each worker is represented by the horizontal line of the worker’s respective color.

In the case of datasets with overall balanced number of class instances, it is observed in Figure 10 that a sufficient increase in the overall accuracy in the range of 3.03% to 4.38% is achieved with 20 queries, for all workers.

### 4.5. Discussion

The above subsections have proven that further customizing the model trained in federation by the 3 workers with AL methods, results in increased accuracy compared with the federated model, for all of the AL dataset bias cases. As such, our methodology is a cost-effective solution for not only improving the overall metrics of the model resulted through the federated procedure, but also for tailoring the model to the participant’s network traffic characteristics. Table 6 below, indicates the average accuracy, precision and F1 percentage difference after training with AL, taking into consideration all the above dataset cases, for each worker.

Notably, although a massive percentage increase did not arise from the experimental results, the customized models are able to classify correctly all instances which belong under the biased category, in all cases, even after 10 queries only. When creating effective IDS to be utilized in critical settings, priority should be given in accurately classifying samples belonging to the worker’s communication characteristics, especially when training models in collaboration, as each worker’s traffic may vary significantly from the rest.

With the above into consideration, we conclude that the fusion of the federated and active learning techniques is a cost-effective, budget-friendly method of cooperative model training, for the creation of robust IDS, able to succeed in the rapid recognition of threats in order to provide the protection needed in critical industrial systems.

## 5. Conclusions

Federated learning is a collaborative training approach which certainly enhances data privacy, however, global models can still improve in terms of performance. To address the high expense of annotating large datasets, active learning is proposed as a personalization method. Specifically, in this paper we have shown that the pairing of federated learning with active learning is able to achieve overall better final model performance with fewer data samples required for personalized training. It is observed that in most cases, 10 to 20 AL queries suffice for creating better, customized local models in a variety of local database settings. Notably, in the case of W3 for 20% AL training dataset bias, the model was able to achieve an increase of 7.07% in accuracy with only 10 AL queries. Furthermore, the average accuracy percentage increase for all dataset bias cases, falls in the range of 1.51% to 6.06%, for all workers, and for all query instances. In addition, even in the cases where metrics show a decrease or in the cases where the increase in accuracy is not significant, the final customized model is able to classify correctly all samples which belong to a class that the local AL dataset is biased towards; in contrast, standalone federated learning is unable to perform as well in this aspect. This indicates that our methodology ensures the security and privacy of the collaborative training process, while also supporting the adaptability of the final local model to the worker’s network traffic, with a minimum labelling budget.

## Figures and Tables

**Table 1:** Evaluation of the global FL model produced after r=3, using Worker 1’s (W1), Worker 2’s (W2) and Worker 3’s (W3) evaluation datasets.

	Accuracy	Precision	F1
W1	0.8013	0.8190	0.8004
W2	0.7508	0.7781	0.7431
W3	0.7037	0.7529	0.7034

**Table 2:** Evaluation of W1, W2 and W3’s personalized models generated with 20% AL dataset bias after Q=10,20,30,40 queries, using their corresponding evaluation datasets. Underlined results mean increased metrics in comparison with the corresponding FL evaluation.

		Accuracy			Precision			F1	
	W1	W2	W3	W1	W2	W3	W1	W2	W3
Q = 10	0.7979	0.7609	0.7744	0.8348	0.8171	0.7188	0.7815	0.7414	0.7191
Q = 20	0.8383	0.7676	0.7272	0.8567	0.7425	0.7658	0.8260	0.7440	0.6914
Q = 30	0.8282	0.7845	0.7609	0.8581	0.8100	0.7070	0.8218	0.7774	0.7053
Q = 40	0.8249	0.7979	0.7744	0.8461	0.8173	0.7184	0.8127	0.7921	0.7195

**Table 3:** Evaluation of W1, W2 and W3’s personalized models generated with 50% AL dataset bias after Q=10,20,30,40 queries, using their corresponding evaluation datasets. Underlined results mean increased metrics in comparison with the corresponding FL evaluation.

		Accuracy			Precision			F1	
	W1	W2	W3	W1	W2	W3	W1	W2	W3
Q = 10	0.8215	0.7441	0.7205	0.7940	0.8192	0.6066	0.8011	0.7062	0.6315
Q = 20	0.8181	0.7946	0.7643	0.8286	0.8419	0.7067	0.8112	0.7777	0.7103
Q = 30	0.8552	0.8350	0.7609	0.8590	0.8471	0.7300	0.8514	0.8295	0.6946
Q = 40	0.8080	0.7777	0.7744	0.8322	0.8051	0.7361	0.8051	0.7694	0.7163

**Table 4:** Evaluation of W1, W2 and W3’s personalized models generated with 70% AL dataset bias after Q=10,20,30,40 queries, using their corresponding evaluation datasets. Underlined results mean increased metrics in comparison with the corresponding FL evaluation.

		Accuracy			Precision			F1	
	W1	W2	W3	W1	W2	W3	W1	W2	W3
Q = 10	0.8350	0.8013	0.7205	0.8190	0.8270	0.6257	0.8113	0.7870	0.6402
Q = 20	0.7777	0.8013	0.7710	0.8025	0.8226	0.7159	0.7610	0.7886	0.7153
Q = 30	0.8282	0.7845	0.7744	0.8333	0.8062	0.7188	0.8227	0.7756	0.7191
Q = 40	0.8249	0.7878	0.7744	0.8300	0.8364	0.7150	0.8198	0.7705	0.7210

**Table 5:** Evaluation of W1, W2 and W3’s personalized models generated with no AL dataset bias after Q=10,20,30,40 queries, using their corresponding evaluation datasets. Underlined results mean increased metrics in comparison with the corresponding FL evaluation.

		Accuracy			Precision			F1	
	W1	W2	W3	W1	W2	W3	W1	W2	W3
Q = 10	0.8148	0.7912	0.6969	0.7800	0.8133	0.7705	0.7849	0.7837	0.6866
Q = 20	0.8316	0.7946	0.7340	0.8469	0.8177	0.8248	0.8224	0.7872	0.7241
Q = 30	0.8080	0.7744	0.7138	0.8214	0.8057	0.7925	0.8048	0.7648	0.6946
Q = 40	0.8316	0.7474	0.7340	0.8474	0.8184	0.76944	0.8245	0.7161	0.7313

**Table 6:** Average difference in percentage of the customized models’ accuracy, precision and F1, considering all dataset balance cases of Section 4.1, Section 4.2, Section 4.3 and Section 4.4.

		Accuracy			Precision			F1	
	W1	W2	W3	W1	W2	W3	W1	W2	W3
Q = 10	1.60%	2.35%	2.43%	−1.20%	4.11%	−7.25%	−0.57%	1.15%	−3.41%
Q = 20	1.51%	3.87%	4.54%	1.47%	2.81%	0.04%	0.48%	3.13%	0.69%
Q = 30	2.85%	4.38%	4.87%	2.40%	3.92%	−1.58%	2.48%	4.37%	0.00%
Q = 40	2.11%	2.69%	6.06%	1.99%	4.12%	−1.82%	1.51%	1.89%	1.86%

## References

[1] Verma A., Ranga V. (2019). Machine learning based intrusion detection systems for IoT applications. Wirel. Pers. Commun..

[2] Kiran S., Kumar U.V., Kumar T.M. A review of machine learning algorithms on IoT applications. Proceedings of the 2020 International Conference on Smart Electronics and Communication (ICOSEC).

[3] Qian B., Su J., Wen Z., Jha D.N., Li Y., Guan Y., Puthal D., James P., Yang R., Zomaya A.Y. (2020). Orchestrating the development lifecycle of machine learning-based IoT applications: A taxonomy and survey. ACM Comput. Surv..

[4] Ma L., Sun B. (2020). Machine learning and AI in marketing – Connecting computing power to human insights. Int. J. Res. Mark..

[5] Bauer W.A., Dubljević V. (2019). AI assistants and the paradox of internal automaticity. Neuroethics.

[6] Amberkar A., Awasarmol P., Deshmukh G., Dave P. Speech recognition using recurrent neural networks. Proceedings of the 2018 International Conference on Current Trends towards Converging Technologies (ICCTCT).

[7] Sodhro A.H., Pirbhulal S., de Albuquerque V.H.C. (2019). Artificial intelligence-driven mechanism for edge computing-based industrial applications. IEEE Trans. Ind. Inform..

[8] Cui L., Qu Y., Gao L., Xie G., Yu S. (2020). Detecting false data attacks using machine learning techniques in smart grid: A survey. J. Netw. Comput. Appl..

[9] Kumar I., Mohd N., Bhatt C., Sharma S.K., Pant M., Kumar Sharma T., Arya R., Sahana B., Zolfagharinia H. (2020). Development of IDS using supervised machine learning. Soft Computing: Theories and Applications.

[10] Kilincer I.F., Ertam F., Sengur A. (2021). Machine learning methods for cyber security intrusion detection: Datasets and comparative study. Comput. Netw..

[11] Gupta A.R.B., Agrawal J. A Comprehensive survey on various machine learning methods used for intrusion detection system. Proceedings of the 2020 IEEE 9th International Conference on Communication Systems and Network Technologies (CSNT).

[12] Radoglou-Grammatikis P., Sarigiannidis P., Efstathopoulos G., Karypidis P.A., Sarigiannidis A. (2020). DIDEROT: An intrusion detection and prevention system for DNP3-based SCADA systems. Proceedings of the 15th International Conference on Availability, Reliability and Security.

[13] Chen Y.C., Mooney V., Grijalva S. Electricity grid cyber-physical security risk assessment using simulation of attack stages and physical impact. Proceedings of the 2020 IEEE Kansas Power and Energy Conference (KPEC).

[14] Langner R. (2011). Stuxnet: Dissecting a cyberwarfare weapon. IEEE Secur. Priv..

[15] Chen T.M., Abu-Nimeh S. (2011). Lessons from Stuxnet. Computer.

[16] Elbir A.M., Coleri S. (2021). A family of hybrid federated and centralized learning architectures in machine learning. arXiv.

[17] Vu L., Nguyen Q.U., Nguyen D.N., Hoang D.T., Dutkiewicz E. (2020). Deep transfer learning for IoT attack detection. IEEE Access.

[18] Drainakis G., Katsaros K.V., Pantazopoulos P., Sourlas V., Amditis A. Federated vs. centralized machine learning under privacy-elastic users: A comparative analysis. In Proceedings of the 2020 IEEE 19th International Symposium on Network Computing and Applications (NCA).

[19] Mothukuri V., Khare P., Parizi R.M., Pouriyeh S., Dehghantanha A., Srivastava G. (2021). Federated learning-based anomaly detection for IoT security attacks. IEEE Internet Things J..

[20] Rahman S.A., Tout H., Talhi C., Mourad A. (2020). Internet of Things intrusion detection: Centralized, on-Device, or federated learning?. IEEE Netw..

[21] Li T., Sahu A.K., Talwalkar A., Smith V. (2020). Federated learning: Challenges, methods, and future directions. IEEE Signal Process. Mag..

[22] Konyushkova K., Sznitman R., Fua P. (2017). Learning active learning from data. arXiv.

[23] Geifman Y., El-Yaniv R. (2019). Deep active learning with a neural architecture search. arXiv.

[24] Truong N., Sun K., Wang S., Guitton F., Guo Y. (2021). Privacy preservation in federated learning: An insightful survey from the GDPR perspective. arXiv.

[25] Dhakal S., Prakash S., Yona Y., Talwar S., Himayat N. Coded federated learning. Proceedings of the 2019 IEEE Globecom Workshops (GC Wkshps).

[26] Li B., Wu Y., Song J., Li T., Zhao L. (2020). DeepFed: Federated deep learning for intrusion detection in industrial cyber-physical systems. IEEE Trans. Ind. Inform..

[27] Nguyen T.D., Marchal S., Miettinen M., Fereidooni H., Asokan N., Sadeghi A.R. DÏoT: A federated self-learning anomaly detection system for IoT. Proceedings of the 2019 IEEE 39th International Conference on Distributed Computing Systems (ICDCS).

[28] Schneble W., Thamilarasu G. Attack detection using federated learning in medical cyber-physical systems. Proceedings of the 28th International Conference on Computer Communications and Networks (ICCCN).

[29] Görnitz N., Kloft M., Rieck K., Brefeld U. Active learning for network intrusion detection. Proceedings of the 2nd ACM Workshop on Security and Artificial Intelligence.

[30] Abe N., Zadrozny B., Langford J. (2006). Outlier detection by active learning. Proceedings of the 12th ACM SIGKDD International Conference on Knowledge Discovery and Data Mining.

[31] Pimentel T., Monteiro M., Veloso A., Ziviani N. (2020). Deep active learning for anomaly detection. arXiv.

[32] Phaladisailoed T., Numnonda T. Machine learning models comparison for bitcoin price prediction. Proceedings of the 2018 10th International Conference on Information Technology and Electrical Engineering (ICITEE).

[33] Gafni T., Shlezinger N., Cohen K., Eldar Y.C., Poor H.V. (2021). Federated learning: A signal processing perspective. arXiv.

